# The importance of Evolutionary Medicine in developing countries: A case for Pakistan's medical schools

**DOI:** 10.1093/emph/eoy004

**Published:** 2018-01-29

**Authors:** Syed Faaiz Enam, Shumaila Hashmi

**Affiliations:** 1 Department of Biomedical Engineering, Duke University, Durham, NC 27708, USA; 2 Greater Manchester Mental Health Trust, Manchester M25 3BL, UK

**Keywords:** Evolutionary Medicine, medical education, Pakistan, developing world, public health, family medicine

## Abstract

Evolutionary Medicine (EM) is a fundamental science exploring why our bodies are plagued with disease and hindered by limitations. EM views the body as an assortment of benefits, mistakes, and compromises molded over millennia. It highlights the role of evolution in numerous diseases encountered in community and family medicine clinics of developing countries. It enables us to ask informed questions and develop novel responses to global health problems. An understanding of the field is thus crucial for budding doctors, but its study is currently limited to a handful of medical schools in high-income countries. For the developing world, Pakistan's medical schools may be excellent starting posts as the country is beset with communicable *and* non-communicable diseases that are shaped by evolution. Remarkably, Pakistani medical students are open to studying and incorporating EM into their training. Understanding the principles of EM could empower them to tackle growing health problems in the country. Additionally, some difficulties that western medical schools face in integrating EM into their curriculum may not be a hindrance in Pakistan. We propose solutions for the remaining challenges, including obstinate religious sentiments. Herein, we make the case that incorporating EM is particularly important in developing countries such as Pakistan and that it is achievable in its medical student body.

## INTRODUCTION

It has long been recognized that evolutionary biology has implications for medical practice. Medicine is currently an ‘unsystematic conglomeration of information and skills gathered from a wide variety of domains without any linking theoretical rationale’ [1]. However, it has been argued since the early 1990s that evolutionary biology could bring cohesion to the many subjects of Medicine [2, 3]. Evolutionary Medicine (EM), also known as Darwinian Medicine, sought to answer the question of why particular aspects of the human body are as they are. The prevailing basic sciences provide proximate explanations for pathology and physiology (e.g. the mechanism of fever after an infection), but EM provides an overarching explanation for both (e.g. why did we evolve to respond with fever?). These explanations then lend insight on how to approach disease (e.g. when and how should fever be treated?). EM thus integrates the various disciplines taught in medical school to promote a unified intellectual understanding. It could grow to be to Medicine what Evolution is to Biology.

Perceiving the symptoms of a patient as a conglomerate of evolutionary benefits, mistakes and compromises may assist physicians in understanding certain pathologies better. It alters how patients are seen: from ‘built’ machines to bodies forged over millennia incorporating different compromises that were viable enough to permit reproduction [4]. These archaic compromises constitute one of the reasons why we suffer disease today [5, 6]. However, inculcation of such percipience in future doctors and scientists requires the inclusion of EM into the medical school curriculum. Such an inclusion has been slow and laden with impediments. Nevertheless, considering the benefits in both knowledge and understanding that EM can provide, these impediments must be surmounted for the benefit of public health.

Myriad public health problems and prevalent diseases today have an evolutionary basis behind them [4]. This includes communicable diseases (CDs) such as viral epidemics, antibiotic resistance and superbugs, and non-communicable diseases (NCDs) like obesity, diabetes, cancer and mental health issues. Unfortunately, developing countries like Pakistan suffer from a dual burden of CDs and NCDs. In fact, NCDs make up half the burden of disease in this sixth most-populous country [7]. Of course, EM is not a clinical practice to treat these diseases. Instead, grounded knowledge in the field can influence practice through controlled trials. Thus the role of EM in the clinic is similar to those of the basic sciences: knowing how action potentials generate has little to do with seeing a patient; however it provides a level of understanding to organize information of disease and form the grounds for researching therapies [4].

Through the lens of EM, physicians, community health workers and scientists can gain insight into why the global population suffers from these diseases. This insight can then guide both scientific and political exploration of novel solutions. One common example is the ‘Mismatch Hypothesis’ based on the fact that biological evolution is very slow and pervasive while cultural evolution within societies is rapid and volatile (the unit of inheritance being the meme, as coined by Richard Dawkins [8]). This has resulted in a conflict between our stone-age genetics and computer-age lives encumbered with surplus food, sedentary occupations, and modern stressors. The implications of this are interesting; chronic, degenerative diseases can be blamed more on our ancestral genotype than on the subtle increase in human longevity. Keeping this perspective in mind could assist research and clinical practice [4].

Because of its immense importance, incorporation of EM into the medical school curriculum in developed countries has gained both credence and pace in the past couple of decades [9]. Major medical education bodies recommended EM to be incorporated in the premedical and medical curricula for the first time in 2009 and these have been extensively reviewed [9–11]. A small portion of medical and premedical schools in the developed world have also incorporated EM into their curricula [12–18] and centers for collaboration have been set up as well [19]. Furthermore, for institutions looking to incorporate EM, multiple groups have proposed syllabi and learning objectives [1, 9, 20]. At least three textbooks on the field have also been published [21–23]. As an example, University of Southern California’s medical school offered EM as a 4-week elective course to 18 final year medical students [12]. Students in the program reported that they would recommend the course and gave high ratings to their perceived gains in understanding, averaging a 4.7 out of 5. Similar study modules in the UK, Switzerland and Australia were equally well-received and the students strongly appreciated the relevance to medicine [16, 17].

However, even with the amplifying call to incorporate EM into the medical curriculum, there have been multiple impediments that need to be addressed. A 2003 questionnaire delivered to medical school deans in North America [13] elucidated that almost half agreed with the importance of EM to physicians. Yet, only 32% reported covering at least half of the core topics of EM; this statistic was also observed in the UK [16]. A 2013 follow-up of the North American study reported a 44% increase in the number of schools covering all topics of EM but only 6% studied EM ‘moderately’ or ‘in depth’ [18]. In all these studies, the most significant hurdles reported were:

- Limited resources (e.g. faculty and books)
- Limited time
- Little importance
- Potential controversy (e.g. religious sentiments)

Nevertheless, given its role in understanding global diseases, it is imperative that EM is incorporated into medical education in the developing world. Unfortunately, as it stands, this has only been tested in the developed world. In this review, a case is presented for why incorporating evolution into medicine is particularly important in a developing country like Pakistan and why it may be easier than intuited. Prevalent diseases in Pakistan are initially discussed alongside relevant insights from EM research. This provides a foundation for the need of EM in medical education in Pakistan. The greatest impediments to incorporating EM into a medical curriculum in the country are then addressed, and the review then identifies why these may not be insurmountable.

## DISEASES IN PAKISTAN AND THEIR EVOLUTIONARY BASIS

Pakistan is a developing, low-middle income country in South Asia with almost 40% of its population of 200 million below the poverty line [24, 25]. People in this stratum have very little or difficult access to quality medical care [26], there is no government-sponsored health insurance, and private insurance covers only those employed [27, 28]. Sparse health infrastructure, rugged mountainous terrains, conflict, terrorism, cultural hindrances and a lack of education and awareness hamper proper medical care especially in rural areas. Urban areas, in contrast, typically possess well-developed private tertiary care centers. However, fees there can be unaffordable and hospitals can be overwhelmed by the number of patients [29]. Communicable, maternal, perinatal and nutritional conditions account for almost 40% of deaths while NCDs surprisingly account for another 50% [7].

To address these issues, the Pakistani populace requires more emphasis on its primary healthcare system (composed of basic health units and rural health centers) and the fields of Family and Community Medicine. These cater to the middle and lower socioeconomic bracket in Pakistan and train physicians for comprehensive healthcare. It is these doctors (and their patients) to whom understanding the evolutionary basis of disease is most relevant.

### Communicable diseases

CDs account for more than one-third of deaths in Pakistan [7] among adults, children and neonates. Pakistan is also one of only two countries in which the poliovirus has yet to be eradicated. Antibiotic resistance and associated epidemics, although a global problem, are likely to have a worse toll on developing nations such as Pakistan, where the cost of treatment can be unaffordable to the patient, a lack of awareness is present, and poor living conditions are common. The nation has also had to deal with outbreaks of dengue, measles and hepatitis frequently and this situation is worsened by changing environmental and climate conditions [30].

Pakistan is host to numerous antibiotic resistant bacteria. For example, extensively drug-resistant strains of *Mycobacterium tuberculosis* have risen from 1.5% in 2006 to 4.5% in 2009 [31]. These strains are resistant to all fluoroquinolones, injectable second-line regimen drugs, and Isoniazid and Rifampin. Bacterial isolates sampled from patients in two major urban cities, Lahore and Islamabad, were multi-drug resistant (including extended-spectrum beta-lactamases) in over 80% of cases [32]. Furthermore, due to inappropriate antibiotic use in public health clinics, pathogens causing community diseases such as urinary tract infections and gastritis are evolving a resistance to fluoroquinolones [33, 34]. Salmonella typhi in Pakistan is also demonstrating resistance to first-line antibiotics [35].

To mitigate the evolution and spread of resistant bacteria, mechanisms need to be better understood by scientists and clinicians, with a stronger understanding of evolutionary biology and ecology. Most studies of resistance have been performed without this foundation [36] and only recently, through empirical evidence, has it been determined that conventional wisdom of ‘complete the full prescription’ may be incorrect [37] while such ideas have been advocated by evolutionary biologists previously [38, 39]. Instead, more studies need to be conducted to truly appreciate the varieties of treatment strategies required to minimize the risk of resistance evolution [40, 41]. While evolution is typically an imperceivably slow process, antibiotic resistance is demonstrable in the lab and within patients, making it appealing to study [42, 43]. Greater emphasis on these processes, and how they serve as a template for natural selection on a larger scale, may be beneficial to budding family physicians [44]. By instilling a deeper understanding of the mechanisms of resistance and the evolution of rapidly evolving pathogens such as viruses, future physicians may be better inspired and equipped to study and handle the health crises that Pakistan and other developing countries are eventually bound to face.

### Non-communicable diseases

NCDs, such as cardio- and cerebrovascular disease, diabetes, kidney disease and cancer are globally rising. However, Pakistan is under a double burden with both infectious and NCDs plaguing the nation and NCDs accounting for half of all deaths [7]. A study in 2013 projects 3.87 million deaths annually in Pakistan by 2025 due to cancer, cardiovascular, and chronic respiratory diseases [45]. To handle this, primary prevention is required and interventions for Pakistan have been suggested [46]. Diet is one of the important risk factors for NCDs [47] and a study in Pakistan demonstrated that in a community almost half did not have adequate intake of fruits and vegetables, more than half were physically inactive and half were also overweight/obese [48]. In fact, obesity has become an epidemic in Pakistan affecting all age groups [49] and the need to tackle both over and under-nutrition to address the rising rate of NCDs is known [50]. Cancer, too, has been rising and although the national registry has never published its data, regional registries and studies demonstrate that lip, oral and larynx cancer is commonest in men (and second commonest in Asia) while breast cancer is commonest in women and also highest in the region [51–55].

A better understanding of the evolutionary mechanisms behind NCDs can benefit medical professionals. The prevailing EM understanding of the rise of NCDs is known as the mismatch hypothesis. This explains that while the human genome evolved thousands of years ago, society has dramatically changed after and the genome has not kept pace (or needed to keep pace) [56–58]. Biological evolution is typically a very slow process while cultural evolution is rapid. The evidence for this exists even now: a study uncovered that modern-day humans in hunter–gatherer communities tend to have a lower incidence of chronic diseases [59]. More strikingly, it also demonstrated that transitioning to a western lifestyle can lead to developing such conditions! Our shift in diet towards carbohydrate-rich foods is an example of the changing culture and it has been shown that South Asians respond to this more negatively than Caucasians [60].

Cancer is supremely relevant in EM as it is a microcosm of natural selection with mutations being selected for increased fecundity of neoplastic cells [61]. Most therapies attempt to cure cancer by lethal drugs that kill sensitive cells but leave a vacuum for resistant cells to proliferate i.e. the cancer evolves. EM models and animal experiments, however, demonstrate that therapies may result in increased patient survival by letting the sensitive population survive and keeping the tumor stable [62]. This knowledge might be especially useful for Pakistani patients as cheaper off-patent drugs could be administered to contain tumor volume. Similarly, the cost of treatment could be further mitigated by preventing cancer (informed through EM principles) [63].

These studies emphasize the need to approach NCDs through the lens of EM. The field has provided unique insights that produce novel hypotheses, models and successful experiments to test these stubborn problems [58, 62, 64, 65]. Regarding cancer in Pakistan, while most patients do end up seeing an oncologist, their first encounter is often with a family physician [66]. However, a multi-center study demonstrated that most of these physicians are unaware of appropriate cancer and palliative care [67]. Health promotion programs through EM can provide family physicians with a better conceptual framework of the disease and inspire critical questions that need to be studied to target various NCDs [44, 68, 69].

## INCORPORATING EM IN PAKISTAN

With the relevance of EM to public health and disease in Pakistan, the need to include it in the medical curricula becomes evident. Pakistan boasts many premier and globally recognized medical institutions with over 170 000 graduates in 2012 [70]. A large number of their medical graduates, however, migrate abroad for residency programs and further practice [71]. Additionally, the emphasis on training doctors has often eclipsed the training of health-care workers whom are required for healthcare promotion and prevention [70]. Indeed, it is these physicians, workers, and additionally researchers, to whom this basic science is most relevant. Although there are many impediments one might perceive germane to Pakistan, these might not be as ardent and may have convincing workarounds. The four most commonly cited difficulties observed in the developed world are reiterated here with a comment on each.

### Argument of limited resources

In Pakistan, there is currently no education on the role of evolution in physiology and disease. However, owing to EM programs in the developed world, textbooks, learning objectives, and syllabi are already published and widely available. Students are also introduced to various concepts of evolution in high school, supported by dedicated chapters in their textbooks [72]. Additionally, the experiences of medical schools on the pedagogical methods to teaching too can be gleaned from. Obtaining these sources would not be difficult for Pakistani medical students as their coursework already promotes internationally published and popular textbooks such as Guyton and Hall’s Medical Physiology and Gray’s Anatomy for Students. Books are also often published with a ‘South Asia Edition’ to be sold at cheaper prices and (unfortunately) textbook piracy is common too.

The resource that requires emphasis is faculty with an interest and education in evolution. These faculties are already present in departments of various universities teaching Biology in Pakistan (such as the LUMS School of Science and Engineering, Quaid-e-Azam University or Karachi University). Furthermore, a 2014 seminar titled ‘Small Variations for Big Changes’ held at the Dow University of Health Sciences and Federal Urdu University of Arts, Science and Technology featured several faculty and students from Pakistan with presentations and posters on evolutionary biology. This was sponsored by an outreach fund from the European Society for Evolutionary Biology. Such seminars and workshops can bring together interested faculty to promote EM in medical schools around Pakistan. There are also numerous Pakistani scientists publishing on evolution and microbiology [73–76].

### Argument of limited time

The common argument for time constraint and a congested curriculum is substantially weaker in Pakistan as subjects outside of the basic sciences are currently included. The medical regulatory body there, the Pakistan Medical and Dental Council (PMDC), mandates the inclusion of Pakistan and Islamic studies in the medical college curriculum [77]. Additionally, a premier medical school has published about their successful 6-week Humanities and Social Sciences courses [78]. These subjects likely have merit in grooming holistic physicians to practice in Pakistan [79, 80]. However, if time can be made for humanitarian subjects, it could also be made for an important basic science like EM. The EM courses that were attempted in the developed world tended to be limited to a period of around four weeks or even as short as 7 half-days spread across a semester.

### Argument of little importance

The importance of EM for Pakistan has been discussed throughout this review. It is exemplified by the relevance of EM in multiple public health diseases and the importance of a strong public health and family medicine program in Pakistan [44]. Healthcare in Pakistan needs to shift from curative to preventive to better assist the population and the economy, and community and family medicine provide holistic care to handle that [29, 81]. Progress is underway to strengthen family medicine programs in Pakistan. Perhaps the study of EM and the conceptual framework it instills will incline more graduates towards community and family medicine and associated research. Additionally, it could make the fields still more intellectually satisfying by promoting critical thinking skills [20]. These arguments are also backed by a 2011 study of two medical schools in Pakistan [82] where a stunning 74% of students surveyed agreed that EM would improve research and 50% agreed on its importance in clinical practice.

### Argument of potential controversy

Religious sentiments may be perceived to be the largest obstacle to incorporating EM in a country like Pakistan. However, these fears may be unfounded and sentiments can be delicately handled or worked around. The first and most commonly quoted study to gauge the acceptance of evolution in Pakistan put the number at 14% among 100 participants in Lahore [83]. However, the sampling methodology is not well described and the statistic may not apply to medical students. Instead, among Pakistani doctors living in the USA, 20 out of 23 accepted evolutionary biology and another two also understood its importance in medicine [84]. Another study of Pakistani teachers demonstrated that 14 out of 18 accepted evolutionary biology (although only 20% accepted human evolution) [85]. Fascinatingly, when Quranic scripture is quoted in some Pakistani high school textbooks, it attempts to support evolutionary biology [86]. More recently, of 43 Muslim Pakistani physicians and medical students, half accepted evolutionary biology and a quarter even agreed that humans have evolved as well (Hameed, S. *et al.* 2017, in review). Only six believed evolution and the notion of a god were contradictory while almost half of their counterparts in Malaysia believed as such, highlighting a uniqueness of Pakistan in the Muslim world.

These data are bolstered by the previously mentioned 2011 study in two medical schools where 300 students were surveyed. Although there initially was a low acceptance rate (mean 58.32) and low knowledge (5.2/10) of evolutionary biology (based on the Measure of Acceptance of Theory of Evolution [87]), 74% agreed that EM would improve research and 50% agreed on its importance in clinical practice. Additionally, 37% agreed that EM ought to be taught in medical school while 16% were undecided—accumulating to more than half the sample. When hindrances were probed, the majority cited a lack of resources and time; only 26% cited difficulties with religious sentiments. These examples show that controversies between evolution and Islam may not be impassable hurdles and that acceptance between the public and medical students may differ. These differences could in part be due to an exposure to biology and evolution in high school, but such correlations need to be explored further. Ultimately, EM pedagogy is geared towards future doctors, health-care workers, and scientists.

For the small proportion of students who may refuse to study EM out of religious beliefs, several solutions can be attempted. First, highlighting the presence of evolutionary thought in Islamic history could be useful. The historic Muslim philosopher Ibn-Khaldun, in his Muqaddimah, proposed ideas to explain the evolution of animals and even implied the relation of humans to apes [85, 88]. Al-Jahiz, in his treatise *The Book of Living Beings*, has also mentioned ideas similar to that of natural selection [89]. Although none of these ideas were a full framework or theory, knowledge of prior acceptance in Islam could make the topic more familiar.

Second, discussing local studies of evolution could make ideas of ‘macro-evolution’ more palatable. One example could be the fascinating start of cetacean evolution in waters in and around modern Pakistan [90]. Pakicetus, the largest of the Pakicetids (fossils of which were found in Pakistan in 1979), was a precursor to modern whales (identified by its inner ear structure) with four legs to wade fresh waters of northern Pakistan and India [91].

Third, it could be stressed that evolution need not be equated with the origin of humans; the two can be studied separately. Based on the data, Pakistani Muslims accept evolution among other organisms (particularly in a theistic context) but stop at humans due to their belief in the divine creation of Adam and Eve. However, EM considers the reasons disease exist ‘after’ humans existed and does not need to address their origins. Of course, human pathology has analogies in the animal kingdom, and thus studying the origin of man may still prove insightful [92]. These analogies could be approached discretely without ever explicitly saying man has a common ancestor with other animals. Consequently, through emphasis on biological evidence, students may understand EM without needing to overtly accept ‘evolution’ itself.

Last, although verses from any religious text can be understood in many ways, certain ‘ayat’ from the Qu’ran may appease the adamantly stubborn: ‘God created you in diverse stages’ (71: 14), ‘Say: Travel in the earth and see how He makes the first creation, then Allah creates the latter creation; surely Allah has power over all things’ (29: 20), and ‘We have made of water everything living, will they not then believe?’ (21:30). While delicately handling the sensitivities of the religiously inclined, through the support of biological and clinical evidence, and examples of evolution and evolutionary thought in their region and religion, the relevance and benefits of EM to their training and servitude to their population can be edified.

## CONCLUSION

It is truly unfortunate that the science that dictated the molding of the human body is not studied by its healers. Even so, as a small step forward, we can surmise at least three conclusions from this review. First, while the call to formalize EM in medical schools has been garnering strong support in the developed world, it has not yet reached the rest of the globe. However, as numerous global health diseases are deeply rooted in evolutionary causes, the teaching of evolution is acutely important in developing countries. Exposing these budding doctors to EM could instill indispensable percipience and critical thinking to solve global health problems. Second, incorporating EM into medical schools in Pakistan can be a real possibility. Limitations that have plagued the western world might not hold as much sway in Pakistan as resources are already present and some curricular time is presently allocated for non-science subjects. Medical schools in the developing world can learn from the trials and tribulations of EM courses abroad. Last, although intuitively it seems conservative religious beliefs are the greatest impediment, in Pakistani medical schools they may not be as large a problem. Additionally, important workarounds could include promoting the Islamic history of evolutionary thought, studying the evolution of animals in the Pakistan region, focusing on the evolution of diseases after the ‘descent of man’, and quoting Quranic scripture. From there, physicians and health-care workers may be better prepared to handle the onslaught of public health issues that developing countries like Pakistan face.

**Conflict of interest:** None declared.

## References

[1] Charlton BG. A syllabus for evolutionary medicine. J R Soc Med 1997;90:397–9. 9290423 10.1177/014107689709000710 PMC1296386

[2] Williams GC, Nesse RM. The dawn of Darwinian medicine. Q Rev Biol 1991;66:1–22. 2052670 10.1086/417048

[3] Nesse RM, Williams GC. Why We Get Sick: The New Science of Darwinian Medicine, 1st ed. New York: Times Books, 1994.

[4] Nesse RM, Stearns SC. The great opportunity: evolutionary applications to medicine and public health. Evol Appl 2008;1:28–48. 25567489 10.1111/j.1752-4571.2007.00006.x PMC3352398

[5] Gluckman PD, Low FM, Buklijas T How evolutionary principles improve the understanding of human health and disease. Evol Appl 2011;4:249–63. 25567971 10.1111/j.1752-4571.2010.00164.x PMC3352556

[6] Gluckman PD, Hanson MA. *Mismatch Why Our Bodies No Longer Fit Our Bodies* USA: Oxford University Press, 2006.

[7] WHO. Global Status Report on Noncommunicable Diseases 2014. http://apps.who.int/iris/bitstream/10665/148114/1/9789241564854_eng.pdf?ua=1 (14 October 2017, date last accessed).

[8] Dawkins R. The Selfish Gene. New York: Oxford University Press, 2013.

[9] Nesse RM, Bergstrom CT, Ellison PT Evolution in health and medicine Sackler colloquium: making evolutionary biology a basic science for medicine. Proc Natl Acad Sci U S A 2010;107:1800–7. 19918069 10.1073/pnas.0906224106 PMC2868284

[10] Labov J. Evolutionary medicine and the medical school curriculum: meeting students along their paths to medical school. Evol Educ Outreach 2011;4:561–6.

[11] Štrkalj G. Darwinian Medicine and "Race"; a Note on Education. Glasnik Etnografskog Instituta SANU 2011;59:231–7.

[12] Abbott A, Abboud G. Evolutionary medicine: a model for medical school introduction. Med Educ 2006;40:471–2. 16635141 10.1111/j.1365-2929.2006.02428.x

[13] Nesse RM, Schiffman JD. Evolutionary biology in the medical school curriculum. Bioscience 2003;53:585–7.

[14] Court JP. Introducing Darwinism to Toronto’s post-1887 reconstituted medical school. Can Bull Med Hist 2011;28:191–212. 21595368 10.3138/cbmh.28.1.191

[15] Wilson DS. Evolution for everyone: how to increase acceptance of, interest in, and knowledge about evolution. PLoS Biol 2005; 3:e364–5. 16336048 10.1371/journal.pbio.0030364 PMC1311567

[16] Downie JR. Evolution in health and disease: the role of evolutionary biology in the medical curriculum. Bee-J 2004;4:1.

[17] Rühli F, Haeusler M, Saniotis A Novel modules to teach evolutionary medicine: an australian and a swiss experience. Med Sci Educ 2016;26:375–81.

[18] Hidaka BH, Asghar A, Aktipis CA The status of evolutionary medicine education in North American medical schools. BMC Med Educ 2015;15:38. 25884843 10.1186/s12909-015-0322-5 PMC4355969

[19] Nunn CL, Wallace I, Beall CM. Connecting evolution, medicine, and public health. Evol Anthropol Issues News Rev 2015;24:127–9. 10.1002/evan.21451 26267433

[20] Graves JL, Reiber C, Thanukos A Evolutionary science as a method to facilitate higher level thinking and reasoning in medical training. Evol Med Public Health 2016;2016:358–68. 10.1093/emph/eow029 PMC5101907 27744353

[21] Gluckman P, Beedle A, Hanson M. *Principles of Evolutionary Medicine*, USA: Oxford University Press, 2009.

[22] Perlman R. Evolution and medicine. Perspect Biol Med 2013;56:167–83. 23974499 10.1353/pbm.2013.0012

[23] Stearns SC, Medzhitov R. Evolutionary Medicine, 1st ed. USA: Sinauer Associates, 2015.

[24] United Nations Development Programme. Multidimensional Poverty in Pakistan. http://www.pk.undp.org/content/dam/pakistan/docs/MPI/Multidimensional%20Poverty%20in%20Pakistan.pdf (14 October 2017, date last accessed).

[25] PBS. *Provisional Summary Results of 6th Population and Housing Census* 2017.

[26] Khowaja K. Healthcare Systems and Care Delivery in Pakistan. J Nurs Adm 2009;39:263–5. 19509600 10.1097/NNA.0b013e3181a96473

[27] Jooma R, Jalal S. Designing the first ever health insurance for the poor in Pakistan–a pilot project. J Pak Med Assoc 2012;62:56–8. 22352104

[28] Raza VF, Iftikhar M, Gulab A Impressions and attitudes of adult residents of Karachi towards a possible public health insurance scheme. J Pak Med Assoc 2017; 67:1460–5. 28924298

[29] Sabzwari S. The case for family medicine in Pakistan. J Pak Med Assoc 2015;65:660–4. 26060167

[30] Khalil AT, Ali M, Tanveer F Emerging viral infections in Pakistan: issues, concerns, and future prospects. Health Security 2017; 15:268–81. 28636447 10.1089/hs.2016.0072

[31] Hasan R, Jabeen K, Ali A Extensively drug-resistant tuberculosis, Pakistan. Emerg Infect Dis 2010;16:1473–5. 20735937 10.3201/eid1609.100280 PMC3294979

[32] Nahid F, Khan AA, Rehman S Prevalence of metallo-beta-lactamase NDM-1-producing multi-drug resistant bacteria at two Pakistani hospitals and implications for public health. J Infect Public Health 2013;6:487–93. 24094832 10.1016/j.jiph.2013.06.006

[33] Abdullah FE, Memon AA, Bandukda MY Increasing ciprofloxacin resistance of isolates from infected urines of a cross-section of patients in Karachi. BMC Res Notes 2012;5:696. 23268858 10.1186/1756-0500-5-696 PMC3536602

[34] Rajper S, Khan E, Ahmad Z Macrolide and fluoroquinolone resistance in Helicobacter pylori isolates: an experience at a tertiary care centre in Pakistan. J Pak Med Assoc 2012; 62:1140–4. 23866399

[35] Khan MI, Soofi SB, Ochiai RL Epidemiology, clinical presentation, and patterns of drug resistance of Salmonella Typhi in Karachi, Pakistan. J Infect Dev Ctries 2012;6:704–14. 23103892 10.3855/jidc.1967

[36] Read AF, Huijben S. Evolutionary biology and the avoidance of antimicrobial resistance. Evol Appl 2009;2:40–51. 25567846 10.1111/j.1752-4571.2008.00066.x PMC3352414

[37] Llewelyn MJ, Fitzpatrick JM, Darwin E The antibiotic course has had its day. BMJ 2017;358:j3418. 28747365 10.1136/bmj.j3418

[38] Read AF, Day T, Huijben S. The evolution of drug resistance and the curious orthodoxy of aggressive chemotherapy. Proc Natl Acad Sci U S A 2011;108:10871–7. 21690376 10.1073/pnas.1100299108 PMC3131826

[39] Pena-Miller R, Laehnemann D, Jansen G When the most potent combination of antibiotics selects for the greatest bacterial load: the smile-frown transition. PLoS Biol 2013;11:e1001540. 23630452 10.1371/journal.pbio.1001540 PMC3635860

[40] Day T, Read AF. Does high-dose antimicrobial chemotherapy prevent the evolution of resistance? PLoS Comput Biol 2016;12:e1004689. 26820986 10.1371/journal.pcbi.1004689 PMC4731197

[41] Kouyos RD, Metcalf CJE, Birger R The path of least resistance: aggressive or moderate treatment? Proc R Soc B Biol Sci 2014;281:20140566. 10.1098/rspb.2014.0566 PMC4211439 25253451

[42] Adam M, Murali B, Glenn NO Epigenetic inheritance based evolution of antibiotic resistance in bacteria. BMC Evol Biol 2008;8:52. 18282299 10.1186/1471-2148-8-52 PMC2262874

[43] Wu Y, Saddler CA, Valckenborgh F Dynamics of evolutionary rescue in changing environments and the emergence of antibiotic resistance. J Theor Biol 2014;340:222–31. 24076261 10.1016/j.jtbi.2013.09.026

[44] Naugler CT. Evolutionary medicine: update on the relevance to family practice. Can Fam Physician 2008;54:1265–9. 18791103 PMC2553465

[45] Jafar TH, Haaland BA, Rahman A Non-communicable diseases and injuries in Pakistan: strategic priorities. Lancet 2013;381:2281–90. 23684257 10.1016/S0140-6736(13)60646-7

[46] Wasay M, Zaidi S, Khan M Non communicable diseases in pakistan: burden, Challenges and way forward for health care authorities. J Pak Med Assoc 2014;64:1218–9. 25831633

[47] Bishwajit G. Nutrition transition in South Asia: the emergence of non-communicable chronic diseases. F1000Research 2015;4:8. 26834976 10.12688/f1000research.5732.1 PMC4706051

[48] Khuwaja AK. KM. Gender differences and clustering pattern of behavioural risk factors for chronic non-communicable diseases: community-based study from a developing country. Chronic Illn 2010;6:163–70. 20444764 10.1177/1742395309352255

[49] Tanzil S, Jamali T. Obesity, an emerging epidemic in Pakistan-a review of evidence. J Ayub Med Coll Abbottabad 2016;28:597–600. 28712244

[50] Iqbal R, Tahir S, Ghulamhussain N. The need for dietary guidelines in Pakistan. J Pak Med Assoc 2017;67:1258–61. 28839315

[51] Qureshi MA, Mirza T, Khan S Cancer patterns in Karachi (all districts), Pakistan: first results (2010–2015) from a Pathology based cancer registry of the largest government-run diagnostic and reference center of Karachi. Cancer Epidemiol 2016;44:114–22. 27566468 10.1016/j.canep.2016.08.011

[52] Khaliq S, Naqvi S, Fatima A. Retrospective study of cancer types in different ethnic groups and genders at Karachi. Springerplus 2013;2:118. 23596561 10.1186/2193-1801-2-118 PMC3625419

[53] Rao SVK, Mejia G, Roberts-Thomson K Epidemiology of oral cancer in Asia in the past decade - An update (2000-2012). Asian Pac J Cancer Prev 2013;14:5567–77. 24289546 10.7314/apjcp.2013.14.10.5567

[54] Bhurgri Y, Faridi N, Kazi LAG Cancer esophagus karachi 1995-2002: epidemiology, risk factors and trends. J Pak Med Assoc 2004;54:345–8. 15449914

[55] Shaukat U, Ismail M, Mehmood N. Epidemiology, major risk factors and genetic predisposition for breast cancer in the Pakistani population. Asian Pac J Cancer Prev 2013;14:5625–9. 24289553 10.7314/apjcp.2013.14.10.5625

[56] Eaton SB, Eaton SB. An evolutionary perspective on human physical activity: implications for health. Comp Biochem Physiol Part A, Mol Integr Physiol 2003;136:153–9. 10.1016/s1095-6433(03)00208-3 14527637

[57] Eaton SB, Konner M. Paleolithic nutrition. A consideration of its nature and current implications. N Engl J Med 1985;312:283–9. 2981409 10.1056/NEJM198501313120505

[58] Wick G, Berger P, Jansen-Dürr P A Darwinian-evolutionary concept of age-related diseases. Exp Gerontol 2003; 38(1-2):13–25. 12543257 10.1016/s0531-5565(02)00161-4

[59] O’Dea K. Westernisation, insulin resistance and diabetes in Australian aborigines. Med J Aust 1991;155:258–64. 1875844 10.5694/j.1326-5377.1991.tb142236.x

[60] Anand SS, Tarnopolsky MA, Rashid S Adipocyte hypertrophy, fatty liver and metabolic risk factors in South Asians: the Molecular Study of Health and Risk in Ethnic Groups (mol-SHARE). PLoS One 2011;6:e22112. 21829446 10.1371/journal.pone.0022112 PMC3145635

[61] Merlo LMF, Pepper JW, Reid BJ Cancer as an evolutionary and ecological process. Nat Rev Cancer 2006;6:924–35. 17109012 10.1038/nrc2013

[62] Gatenby RA, Silva AS, Gillies RJ Adaptive therapy. Cancer Res 2009;69:4894–903. 19487300 10.1158/0008-5472.CAN-08-3658 PMC3728826

[63] Maley CC, Szabo E, Reid BJ. Somatic evolution in neoplastic progression and cancer prevention In: Pre-Invasive Disease: Pathogenesis and Clinical Management. New York, NY: Springer, 2011, 111–27.

[64] Wells JCK, Nesse RM, Sear R Evolutionary public health: introducing the concept. Lancet 2017;390:500–9. 28792412 10.1016/S0140-6736(17)30572-X

[65] Goymer P. Natural selection: the evolution of cancer. Nature 2008;454:1046–8. 18756229 10.1038/4541046a

[66] Yusuf A. Cancer care in Pakistan. Jpn J Clin Oncol 2013;43:771–5. 23749983 10.1093/jjco/hyt078

[67] Shaikh AJ, Khokhar NA, Raza S Knowledge, attitude and practices of non-oncologist physicians regarding cancer and palliative care: a multi-center study from Pakistan. Asian Pac J Cancer Prev 2008;9:581–4. 19256742

[68] Eaton SB, Strassman BI, Nesse RM Evolutionary health promotion. Prev Med (Baltim) 2002;34:109–18. 10.1006/pmed.2001.0876 11817903

[69] Eaton SB, Cordain L, Eaton SB. An evolutionary foundation for health promotion. World Rev Nutr Diet 2001;90:5–12. 11545045 10.1159/000059815

[70] Ghaffar A, Zaidi S, Qureshi H, Hafeez A. Medical education and research in Pakistan. Lancet 2013;381:2234–6. 23684262 10.1016/S0140-6736(13)60146-4

[71] Zaidi SA. Undergraduate medical education in underdeveloped countries: the case of Pakistan. Soc Sci Med 1987;25:911–9. 3317891 10.1016/0277-9536(87)90261-9

[72] Asghar A, Hameed S, Farahani NK Evolution in Biology Textbooks: A Comparative Analysis of 5 Muslim Countries. Religion & Education 2014;41:1–15.

[73] Abbasi AA. Evolution of vertebrate appendicular structures: Insight from genetic and palaeontological data. Dev Dyn 2011;240:1005–16. 21337665 10.1002/dvdy.22572

[74] Akram M, Fatima Z, Purdy MA Introduction and evolution of dengue virus type 2 in Pakistan: a phylogeographic analysis. Virol J 2015;12:148. 26395339 10.1186/s12985-015-0371-8 PMC4579586

[75] Enam SF, Qureshi H, Qureshi SA Sampling bacterial biodiversity from a highly contaminated stream flowing through a densely populated urban area in Karachi. J Ayub Med Coll Abbottabad 2011;23:147–51. 24800368

[76] Siddiqui R, Khan NA Acanthamoeba is an evolutionary ancestor of macrophages: a myth or reality? Exp Parasitol 2012;130:95–7. 22143089 10.1016/j.exppara.2011.11.005

[77] Pakistan Medical & Dental Council. Curriculum of M.B.B.S. http://www.pmdc.org.pk/LinkClick.aspx?fileticket=EKfBIOSDTkE%3D (14 October 2017, date last accessed).

[78] Ghias K, Khan KS, Ali R Stretching the boundaries of medical education A case of medical college embracing humanities and social sciences in medical education. Pak J Med Sci 2016;32:911–6. 27648038 10.12669/pjms.324.10237 PMC5017101

[79] Khan K, Azfar S, Ali R A workshop on relevance of teaching humanities and social sciences in health professions education. Education in Medicine Journal 2014;6:77.

[80] Wachtler C, Lundin S, Troein M. Humanities for medical students? A qualitative study of a medical humanities curriculum in a medical school program. BMC Med Educ 2006;6:16. 16519815 10.1186/1472-6920-6-16 PMC1448176

[81] Pappas G, Glasser JH, Akhter M Healing the schism: medicine and public health in Pakistan. Educ Health (Abingdon) 2008;21:173. 19034838

[82] Yousuf A, bin Daud MA, Nadeem A. Awareness and acceptance of evolution and evolutionary medicine among medical students in Pakistan. Evol Educ Outreach 2011;4:580–8.

[83] Hassan R. On being religious: patterns of religious commitment in Muslim societies. Muslim World 2007;97:437–78.

[84] Everhart D, Hameed S. Muslims and evolution: a study of Pakistani physicians in the United States. Evol Educ Outreach 2013;6:2.

[85] Asghar A. Canadian and Pakistani Muslim teachers’ perceptions of evolutionary science and evolution education. Evol Educ Outreach 2013;6:10.

[86] Asghar A, Wiles JR, Alters B. The origin and evolution of life in Pakistani High School Biology. J Biol Educ 2010;44:65–71.

[87] Rutledge ML, Warden MA. The Development and Validation of the Measure of Acceptance of the Theory of Evolution Instrument. Sch Sci Math 1999;99:13–8.

[88] Khaldun I, Rosenthal F *The Muqaddimah: An Introduction to History* USA: Princeton University Press, 1969.

[89] Stott R. Darwin’s Ghosts: The Secret History of Evolution. USA: Spiegel & Grau, 2012.

[90] Thewissen J, Williams GM. The early radiations of cetacea (mammalia): evolutionary pattern and developmental correlations. Annu Rev Ecol Syst 2002;33:73.

[91] Thewissen JGM, Williams EM, Roe LJ Skeletons of terrestrial cetaceans and the relationship of whales to artiodactyls. Nature 2001;413:277–81. 11565023 10.1038/35095005

[92] Natterson-Horowitz B, Bowers K. Zoobiquity: The Astonishing Connection Between Human and Animal Health. UK: Vintage Books, 2013

